# Comparative Gene Expression Patterns of Two EcobNPV Strains in *Ectropis grisescens* Revealed by Transcriptome Analysis

**DOI:** 10.3390/microorganisms14071599

**Published:** 2026-07-22

**Authors:** Xinxin Zhang, Yang Mei, Guoqing Chen, Qiang Xiao, Meijun Tang, Guozhong Feng

**Affiliations:** 1 State Key Laboratory of Rice Biology and Breeding, China National Rice Research Institute, Hangzhou 311401, China; zhangxinxin_19@163.com (X.Z.); chenguoqing@caas.cn (G.C.); 2 Key Laboratory of Tea Quality and Safety Control, Tea Research Institute, Chinese Academy of Agricultural Sciences, Hangzhou 310008, China; xqtea@tricaas.com; 3 College of Plant Protection, Jilin Agricultural University, Changchun 130118, China; meiyang@jlau.edu.cn

**Keywords:** *Ectropis obliqua* nucleopolyhedrovirus, transcriptomics, time-course analysis, viral gene expression, virulence

## Abstract

*Ectropis obliqua* nucleopolyhedrovirus (EcobNPV) is an important biocontrol agent against *Ectropis obliqua* and *E. grisescens*. A previously isolated strain, EcobNPV-QF4, exhibits significantly higher virulence than the original strain EcobNPV-QV, yet the molecular basis for this difference remains unclear. Leaf-dipping bioassays demonstrated that EcobNPV-QF4 caused significantly higher larval mortality than EcobNPV-QV from 10 dpi onward, reaching 97.9% versus 33.8% at 16 dpi, and higher pupal mortality (100% versus 47.3%), confirming its superior virulence across both larval and pupal stages. To investigate the transcriptional dynamics underlying virulence variation, we performed a comparative time-course transcriptomic analysis of *E. grisescens* infected with either strain at 0, 2, 6, 12, 24, 36, and 48 h post-infection. The reliability of the RNA-seq data was validated by qRT-PCR for selected expressed genes. The results showed that, relative to EcobNPV-QV, the EcobNPV-QF4 strain exhibits a transcriptional strategy characterized by comprehensive and accelerated activation: the transcriptional initiation of its functional modules is globally advanced by approximately 6 h compared with EcobNPV-QV, and genes associated with midgut escape, virion assembly, and viral DNA replication are preferentially and coordinately expressed, resulting in a more synchronized transcriptional profile. Based on these findings, we hypothesize that the enhanced virulence of EcobNPV-QF4 is not attributable to a single factor but rather reflects a synergistic, multitiered acceleration of transcriptional progression that may compress the infection cycle and enhance viral dissemination efficiency. These findings provide critical insights into the molecular mechanisms that may underlie baculovirus virulence and provide a basis for future mechanistic studies.

## 1. Introduction

*Ectropis obliqua* nucleopolyhedrovirus (EcobNPV) is a member of the Baculoviridae family. It was first identified in tea plantations in Anhui Province, China, in 1977 [1]. Extensive research on the formulation and application technologies of EcobNPV has been conducted, and it is now widely utilized for pest control in tea gardens across China [2]. In 2017, a highly virulent strain, EcobNPV-QF4, was isolated from the original strain EcobNPV-QV. Bioassays demonstrated that the mortality rate of *Ectropis grisescens* treated with EcobNPV-QF4 reached 88.2% on the 12th day post-infection, compared to 58.2% for the original strain, representing a 51.5% relative increase in lethality [3]. The median lethal time (LT_50_) for EcobNPV-QF4 was 13.9 days, which was 1.5 days shorter than that of EcobNPV-QV (15.4 days) [3,4]. To elucidate the molecular basis for this difference in virulence, whole-genome sequencing of both strains was performed. The genome size of EcobNPV-QF4 is 132,358 bp, which is 315 bp larger than that of EcobNPV-QV (132,043 bp). Comparative genomic analysis revealed an inversion in the *hr1*–*hr3* region of EcobNPV-QF4. Additionally, the gene encoding the small subunit of ribonucleotide reductase (*rr2*) is duplicated in EcobNPV-QF4 [5]. Although whole-genome sequencing has revealed genomic structural differences between the two strains, these static genomic features alone are insufficient to explain the observed variation in virulence during the dynamic course of infection. Deciphering this mechanism hinges on understanding how these genomic differences influence the spatiotemporal regulation and expression patterns of viral gene transcription. Therefore, building upon genomic characterization, a systematic investigation of the transcriptional dynamics of viral genes is a crucial step to bridge the knowledge gap between genotypic variation and virulence differences.

Baculoviruses possess two virion forms: budded virus (BV) and occlusion-derived virion (ODV) [6]. Infection is typically initiated upon oral ingestion of occlusion bodies (OBs). Following dissolution of the OB protein matrix in the alkaline midgut lumen, ODVs are released to infect midgut epithelial cells. Upon entry, viral nucleocapsids are transported to the nucleus, where viral DNA replication is subsequently initiated and proceeds predominantly during the late phase of infection [7,8]. This intricate life cycle necessitates a tightly regulated transcriptional cascade, conventionally divided into immediate early, early, late, and very late, each governed by specific sets of genes [9,10]. In this program, immediate early genes are transcribed by host RNA polymerase II without requiring prior viral protein synthesis, setting them apart from the early, late, and very late phases, which operate through a hierarchical transcriptional cascade driven by successively expressed viral regulatory factors. For instance, the temporal shift from BV formation, which is required for systemic dissemination, to the occlusion of ODVs into OBs exemplifies how this cascade coordinates the expression of genes governing nucleocapsid assembly, nuclear egress, and the structural remodeling underlying the BV-to-ODV phenotypic switch [11,12,13,14]. Later in infection, a subset of nucleocapsids is retained within the nucleus to be enveloped and occluded into new OBs, a process that requires the precisely timed expression of very late genes encoding *polh* and other occlusion-associated proteins [15]. Given that the kinetics and magnitude of this transcriptional cascade directly determine viral replication efficiency; dissemination capacity; and ultimately, virulence, a detailed understanding of the temporal expression patterns of viral genes is fundamental to elucidating baculovirus pathogenesis.

Transcriptome analysis has emerged as a powerful approach to delineate temporal viral gene expression programs in diverse host systems. For instance, transcriptome analysis of Cydia pomonella granulovirus (CpGV-M) provided a transcriptional landscape of CpGV infection and revealed phase-specific transcription patterns associated with viral DNA synthesis and infectious virion production [16]. Similarly, comparative transcriptomic analysis of *Autographa californica* multiple nucleopolyhedrovirus (AcMNPV) infection between a permissive cell line and the larval midgut identified tissue-specific differences in the expression of genes associated with BV production and midgut escape [17]. While these studies have established transcriptomic approaches as effective tools for dissecting baculovirus gene expression programs, the transcriptional dynamics and functional landscape of EcobNPV remain poorly characterized and warrant systematic investigation. This knowledge gap directly limits our ability to connect the genomic structural variations identified between EcobNPV-QF4 and EcobNPV-QV to their functional consequences during infection. In this study, we performed a comparative time-course transcriptomic analysis of *E. grisescens* following oral infection with each strain. Compared with EcobNPV-QV, EcobNPV-QF4 exhibited coordinated expression of a broader set of functionally related genes, with its transcriptional program advanced by approximately 6 h. We further identified differentially regulated genes between the two strains. Among them is the key gene *fibroblast growth factor* (*fgf*), which mediates viral midgut penetration and systemic spread, together with the DNA replication gene *late expression factor 3* (*lef3*) and virion assembly gene *polyhedrin* (*polh*). We propose that the enhanced virulence of EcobNPV-QF4 correlates with a synergistic, multi-level acceleration of the transcriptional program, potentially compressing the infection cycle and enhancing viral dissemination efficiency.

## 2. Materials and Methods

### 2.1. Insect Rearing and Viral Strains

*E. grisescens* larvae were originally collected from tea plantations in Yueqing (120.98° E, 28.11° N), Wenzhou City, Zhejiang Province, China. The insect colony was maintained under laboratory conditions at 25 ± 1 °C with 70–80% relative humidity and a 12 h light: 12 h dark photoperiod. Larvae were fed daily with fresh, uncontaminated tea leaves and were reared for a minimum of three successive generations prior to experimental use. Two EcobNPV strains, designated EcobNPV-QV and EcobNPV-QF4, were obtained from the virus stock collection of the Tea Research Institute, Chinese Academy of Agricultural Sciences, where they were preserved at 4 °C.

### 2.2. Virulence Bioassay

The virulence of the two EcobNPV strains against *E. grisescens* was determined using 2nd-instar larvae with the leaf-dipping method [3]. Stock suspensions of each virus were diluted to 1.25 × 10^6^ OB (occlusion body)/mL. Fresh tea leaves collected from the field were immersed in the virus suspension, air-dried, and then fed to larvae. After a 3-day feeding period on treated leaves, larvae were transferred to uncontaminated foliage and reared until pupation. Each treatment consisted of approximately 30 larvae, with three biological replicates, and mortality was recorded daily. A control group received distilled water under otherwise identical conditions.

### 2.3. Infection and Sampling

Viral suspensions were prepared by diluting stock OBs with distilled water to a final concentration of 2 × 10^8^ OB/mL. Fresh tea leaves were cut into 9 mm diameter discs using a circular punch, and 10 μL of the viral suspension was applied to the abaxial surface of each disc. Discs were allowed to air dry in a fume hood before being placed individually into wells of 24-well bioassay plates (Thermo Fisher Scientific, Waltham, MA, USA). Third-instar *E. grisescens* larvae of similar size were selected and deprived of food for 8 h prior to infection. Each larva was then transferred to a well containing a virus-treated leaf disc and allowed to feed until the entire disc was consumed. Only larvae that completely ingested the disc were retained for further sampling. The time point at which the disc was fully consumed was designated 0 h post infection (hpi).

To capture the early transcriptional dynamics of viral infection, sampling time points were selected based on prior evidence that viral DNA copy numbers fluctuate substantially within the first 48 hpi [18]. Accordingly, larvae were collected at 0, 2, 6, 12, 24, 36, and 48 hpi. At each designated time point, four biological replicates were collected per viral strain, with each replicate consisting of three pooled larvae. Three additional biological replicates of mock-infected larvae, each pooled from three individuals, served as controls. Samples were immediately flash-frozen in liquid nitrogen for 5 min and stored at −80 °C until RNA extraction.

### 2.4. RNA Extraction and Transcriptome Sequencing

Total RNA was extracted from each larval sample using TRIzol reagent (Invitrogen, Carlsbad, CA, USA) following the manufacturer’s instructions. RNA integrity and concentration were assessed using a Bioanalyzer 2100 system (Agilent Technologies, Santa Clara, CA, USA) and a Qubit 2.0 Fluorometer (Thermo Fisher Scientific, Waltham, MA, USA). Samples with RNA integrity number (RIN) values ≥ 7.0 were used for library construction. mRNA was enriched from total RNA using oligo (dT)-conjugated magnetic beads, and cDNA libraries were prepared with the NEBNext^®^ Ultra™ II RNA Library Prep Kit (New England BioLabs, Ipswich, MA, USA) according to the manufacturer’s protocol. Briefly, first-strand cDNA was synthesized using random hexamer primers, followed by second-strand synthesis. The double-stranded cDNA was then subjected to end repair, A-tailing, and adapter ligation. Library fragments were purified and size-selected using AMPure XP beads (Beckman Coulter, Brea, CA, USA). Library quality was evaluated by Qubit quantification, Bioanalyzer profiling, and quantitative real-time PCR. Paired-end sequencing (150 bp reads) was performed on an Illumina NovaSeq 6000 platform (Illumina, San Diego, CA, USA) by Novogene Co., Ltd. (Beijing, China).

### 2.5. Read Processing and Transcript Abundance Quantification

Raw sequencing reads in FASTQ format were preprocessed using fastp v.0.19.7 with default parameters to remove adapter sequences [19], low quality bases, and reads containing ambiguous ‘N’ bases. Clean reads were aligned to a combined reference genome comprising the *E. grisescens* official gene set (OGS) [20] and the complete genome sequences of both EcobNPV strains. Read alignment was performed using HISAT2 v.2.2.1 with default settings [21]. Gene expression levels were quantified using StringTie v.2.1.4 [22] and expressed as transcripts per million (TPM) to normalize for gene length and sequencing depth. TPM values were calculated based on the number of mapped reads and gene length, enabling direct comparison of expression levels across samples and time points. Transcriptomic data supporting the conclusions of this article is available at InsectBase (http://v2.insect-genome.com/econpv, accessed on 10 December 2025).

### 2.6. Genome-Wide Read Coverage Analysis of Viral Genomes

To evaluate the genome-wide distribution of viral reads, clean paired-end RNA-seq reads from each sample were mapped separately to the reference genomes of EcobNPV-QV and EcobNPV-QF4. Reference genome indexes were first built using Bowtie2 v2.5.5. Read alignment was performed with Bowtie2 using the --very-sensitive-local mode. The resulting SAM files were converted to BAM format, sorted, and indexed using SAMtools v1.23.1. Mapping statistics were summarized using samtools flagstat and samtools idxstats.

Per-base read depth across each viral genome was calculated using samtools depth with zero-coverage positions retained. The command option -aa was used to output all genomic positions, including positions with zero coverage, and -d 0 was used to remove the maximum depth limitation. For each sample, genome-wide coverage statistics were calculated, including mean depth, median depth, maximum depth, and the proportions of genomic positions covered by at least 1×, 5×, and 10× reads.

To visualize the coverage distribution along the viral genome, per-base depth values were summarized into fixed-size genomic windows. The mean depth within each window was calculated and biological replicates within the same treatment group were averaged. Genome-wide coverage profiles were plotted along viral genome coordinates using *R* (version 4.3.0).

### 2.7. Comparative Analysis of Viral Gene Expression Dynamics

Primary rank-based analysis. Given the distinct characteristics of viral transcriptomes, particularly the low viral read proportion (<2% of total reads), asynchronous infection across tissues, and limited number of open reading frames (ORFs) (*n* = 131), conventional differential expression tools relying on high mapping rates and synchronous infection are challenging. Therefore, we adopted a rank-based analytical strategy adapted from previous studies [17]. For each sample, all 131 predicted viral ORFs were sorted by their TPM values and assigned a rank from 1 (lowest expression) to 131 (highest expression). To identify differentially expressed genes between strains at each time point, we compared the rank of each ORF between EcobNPV-QV and EcobNPV-QF4 infected samples. A gene was considered differentially expressed if its rank differed by more than 20 positions and significantly differentially expressed if the rank difference exceeded 30 positions. Genes with TPM ≤ 1 were considered unexpressed and excluded from functional gene counts.

To compare global transcriptional timing between the two strains, we performed a time-shift correlation analysis using all common viral genes. For each candidate shift of −12, −6, 0, 6, or 12 h, the EcobNPV-QF4 expression time series (log_2_(TPM + 1) at 0, 6, 12, 24, 36, 48 hpi) was aligned to the EcobNPV-QV series. For a given shift, only genes with at least two valid paired time points (i.e., non-missing values in both strains at the corresponding aligned time points) were included in the analysis. Pearson correlation coefficients were computed between paired time points for each eligible gene and then averaged across all eligible genes. The shift yielding the highest mean correlation was defined as the optimal shift. No interpolation was applied; therefore, the minimum resolvable shift is 6 h, constrained by the sampling interval. To evaluate the statistical robustness of the optimal shift, we performed three additional analyses. First, for each individual gene, we identified the shift that yielded its maximum Pearson correlation coefficient; the distribution of these gene-level optimal shifts was then summarized. Second, bootstrap resampling with replacement was applied at the gene level (1000 iterations). In each iteration, genes were randomly sampled with replacement, and the optimal shift was recalculated as the shift yielding the highest mean correlation across the resampled gene set. The frequency with which each shift was selected as optimal across all bootstrap iterations was recorded, and 95% confidence intervals for the mean correlation at each shift were estimated using the 2.5th and 97.5th percentiles of the bootstrap distribution. Third, a paired *t*-test was performed to compare the correlation coefficients of individual genes between the optimal shift (−6 h) and the synchronous alignment (0 h), including only genes with valid correlation estimates at both shifts. All statistical analyses were performed using *R* (version 4.3.0).

Supplementary statistical validation using DESeq2 v1.42.0. To complement the rank-based approach with parametric testing and to provide effect size estimates (log_2_ fold changes) and adjusted *p*-values for specific time points with sufficient read depth, we additionally performed DESeq2 analysis on the viral ORF-level raw counts. Raw counts were generated from BAM files using featureCounts v2.1.1 based on strain-specific annotations. One-to-one orthologous ORFs between the two strains were used for cross-strain comparison. DESeq2 analysis was performed separately for each time point with adequate viral read abundance, using viral strain as the explanatory variable. Low-count ORFs and samples with zero assigned reads were excluded from formal testing. Wald test *p*-values and Benjamini–Hochberg adjusted *p*-values were calculated. Genes with adjusted *p* < 0.05 and |log_2_FoldChange| ≥ 1 were considered statistically supported. For genes with extremely low abundance where adjusted *p*-values were unavailable due to independent filtering, these were retained and flagged in the Table S6. Notably, while the rank-based analysis remains our primary inference tool for sparse asynchronous infection data, the DESeq2 results are presented as supportive evidence where sequencing depth permits.

### 2.8. Validation of Key Differentially Expressed Genes via Quantitative Real-Time PCR

Gene expression levels of the viral genes *fgf*, *immediate early gene 0 (ie0)*, *polh*, and *per os infectivity factor 3* (*pif3*) were validated by quantitative real-time PCR (qRT-PCR) on a CFX96 real-time system (Bio-Rad, Hercules, CA, USA). The primer sequences used in this study were synthesized by Zhejiang Shangya Biotechnology (Hangzhou, China) and are listed in Table 1. Each 20 μL reaction contained 10 μL ChamQ Universal SYBR qPCR Master Mix (Vazyme, Nanjing, China), 1 μL each primer (10 μM), 6 μL nuclease-free water, and 2 μL cDNA template. The cycling protocol was: 95 °C for 3 min, followed by 40 cycles of 95 °C for 10 s and 60 °C for 20 s. Melting curve analysis (60–95 °C, 0.5 °C increment per 5 s) was performed to confirm amplification specificity. Each sample was measured in three technical replicates per biological replicate (four biological replicates per time point). GAPDH was used as the housekeeping gene for normalization. Cycle threshold (Ct) values were exported from Bio-Rad CFX Manager 3.0. Relative expression levels were calculated using the 2^−ΔΔCt^ method (where ΔCt = Ct_target − Ct_GAPDH). Time points analyzed were 12, 24, 36, and 48 hpi.

## 3. Results

### 3.1. Virulence of Two EcobNPV Strains

**Figure 1:**
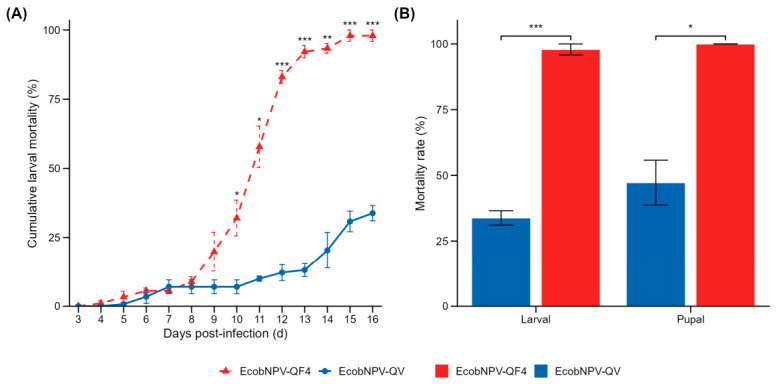
Virulence of two EcobNPV strains against *E. grisescens*. (**A**) Cumulative larval mortality over time. Data points represent mean ± SEM of three replicates. Significant differences between strains at each time point were assessed by Welch’s *t*-test (* *p* < 0.05, ** *p* < 0.01, *** *p* < 0.001). (**B**) Larval mortality at 16 dpi and pupal mortality of the two strains. Bars show mean ± SEM of three replicates. Significance brackets indicate comparisons between strains within each stage (Welch’s *t*-test: * *p* < 0.05, *** *p* < 0.001).

The virulence of EcobNPV-QV and EcobNPV-QF4 against *E. grisescens* was compared using the leaf-dip bioassay. At each time point, mortality in the EcobNPV-QF4 group exceeded that in the EcobNPV-QV group (Figure 1A). Significant differences between the two strains emerged from 10 days post-infection (dpi) (*p* < 0.05) and persisted through 16 dpi (*p* < 0.001). By 16 dpi, larval mortality reached 33.8% ± 2.7% for EcobNPV-QV versus 97.9% ± 2.1% for EcobNPV-QF4 (*p* < 0.001) (Figure 1B). Among pupated individuals, pupal mortality was 47.3% ± 8.5% in the EcobNPV-QV group, whereas all EcobNPV-QF4 infected pupae failed to emerge, resulting in 100% pupal mortality across all replicates (*p* < 0.05). These results demonstrate that EcobNPV-QF4 possesses substantially higher virulence against *E. grisescens* than EcobNPV-QV, as reflected in both larval mortality kinetics and pupal lethality.

### 3.2. Global Transcriptional Profiles of the Two EcobNPV Strains in E. grisescens

To profile the transcriptional dynamics, RNA sequencing was performed on 59 samples of third-instar *E. grisescens* collected at 0, 2, 6, 12, 24, 36, and 48 hpi, as well as uninfected control larvae (CK). Sequencing yielded approximately 410.72 Gb of high-quality data (Q20 > 96%, Q30 > 91%), with uniform GC content (Table S1). Clean reads from each biological replicate were aligned to the reference genomes of EcobNPV-QV (GenBank: OK181759), EcobNPV-QF4 (GenBank: MZ394738), and the host *E. grisescens* (BioProject: PRJNA660825). Genome-wide read coverage distributions across the infection time course were further examined for both strains (Figure S1), illustrating the dynamic changes in viral genomic occupancy and transcriptional activity during infection.

**Figure 2:**
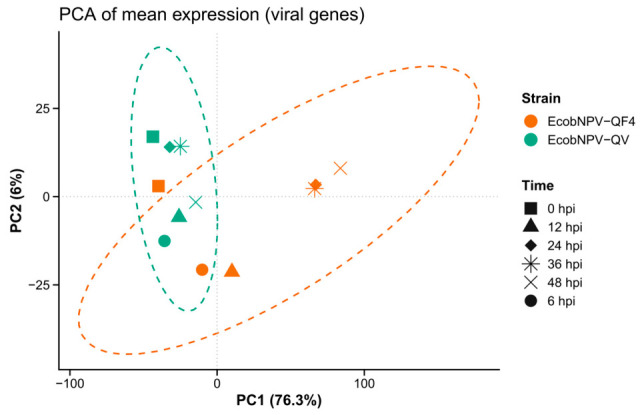
PCA of viral gene expression profiles in *E. grisescens* infected with EcobNPV-QV and EcobNPV-QF4. PCA was performed using the mean expression values of viral genes (log_2_(TPM + 1)) for each strain-time combination, averaged from four biological replicates per time point (0, 2, 6, 12, 24, 36, and 48 hpi). Percentages of variance explained by each principal component are shown in parentheses. PC1 primarily separates samples by infection stage, whereas PC2 captures strain-specific variation, ellipses denote 95% confidence intervals for each strain.

To delineate the global transcriptional dynamics of the two EcobNPV strains during infection, principal component analysis (PCA) was performed on the mean log_2_(TPM + 1) values of viral genes for each strain–time combination, averaged from four biological replicates (Figure 2). The PCA results based on individual replicates are provided in Figure S2. PC1 (76.3%) predominantly captured the temporal progression of infection, with both strains exhibiting a coordinated shift toward positive PC1 values over time. From 12 hpi onward, EcobNPV-QF4 consistently occupied higher PC1 coordinates than EcobNPV-QV at corresponding time points, indicating that EcobNPV-QF4 reached equivalent transcriptional states earlier than EcobNPV-QV. Strain-specific divergence became noticeable from 12 hpi onward and was primarily captured along PC2 (6%). Notably, the tight clustering of individual biological replicates observed in Figure S2 confirmed the high reproducibility of the dataset. This was further substantiated by the sample-to-sample Pearson correlation heatmap (Figure S3), which revealed intra-group correlations among biological replicates and clear segregation between strains and time stages, collectively corroborating the reliability of the transcriptomic data.

Consistent with this pattern, temporal analysis of viral read abundance revealed that the proportion of viral transcripts remained low for both strains at early time points (0–6 hpi), though EcobNPV-QF4 consistently accounted for a higher percentage than EcobNPV-QV (Table S1). An increase occurred at 12 hpi, with viral reads reaching 0.00390% for EcobNPV-QV and 0.06733% for EcobNPV-QF4. The disparity peaked at 48 hpi, where EcobNPV-QF4 achieved a maximum of 1.68836% viral reads, compared to 0.00134% for EcobNPV-QV. The proportion of viral reads in EcobNPV-QF4 was significantly higher than that in EcobNPV-QV from 24 hpi (Mann–Whitney U test, *p* < 0.05). No viral reads were detected in uninfected control larvae. Since viral reads were undetectable at 2 hpi, this time point was omitted from downstream analyses.

### 3.3. Transcriptional Dynamics of EcobNPV-QV in E. grisescens

We first characterized the transcriptional program of the EcobNPV-QV strain in detail. Viral gene expression profiles were analyzed at 0, 6, 12, 24, 36, and 48 hpi (Table S2). The overall expression levels of EcobNPV-QV genes during infection are shown in Figure S4. Based on the mean TPM values calculated for each ORF, the transcriptional patterns at 6 and 12 hpi were similar, as were those from 24 to 48 hpi. Notably, genes highly expressed at 6 and 12 hpi showed decreased expression by 24 hpi.

**Figure 3:**
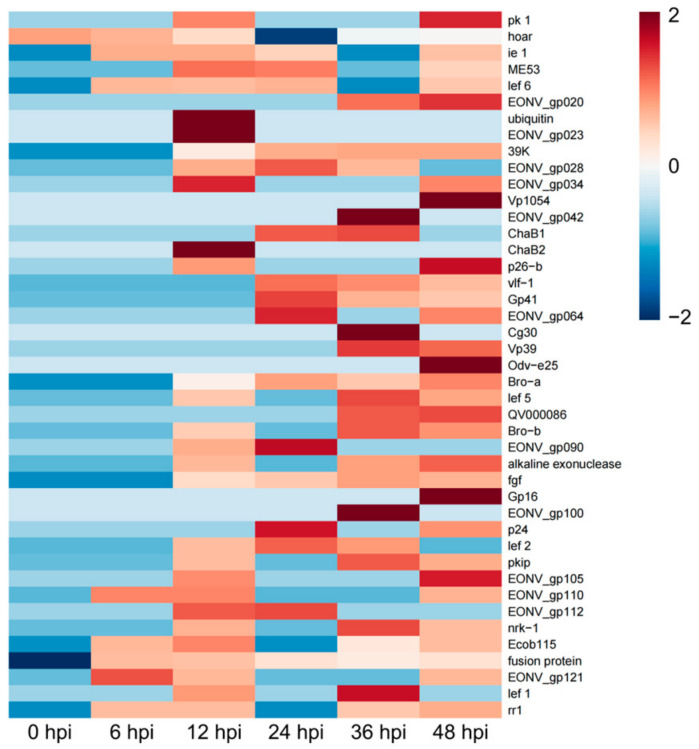
Heatmap of highly expressed genes (TPM > 20,000) across different time points in the EcobNPV-QV. The heatmap depicts the expression dynamics (measured in TPM) of viral genes with peak expression exceeding 20,000 TPM at any sampled time point (0, 6, 12, 24, 36, 48 hpi) during infection of *E. grisescens* by the EcobNPV-QV strain. Expression values (log_2_(TPM + 1)) were Z-score normalized across time points for each gene.

Genes with TPM values exceeding 20,000 were defined as highly expressed [16]. For visualization, the selected genes were log_2_-transformed and row-wise Z-score normalized, and their complete temporal expression profiles across all six time points (0, 6, 12, 24, 36, 48 hpi) were displayed as heatmaps. Those identified as highly expressed in at least one time point are presented in a heatmap (Figure 3), and a detailed list is provided in Table S3. As shown in Figure 3, *ie1* and *late expression factor 6* (*lef6*) exhibited coordinated high expression, beginning at 6 hpi, persisting until 24 hpi, and showing elevated expression again at 48 hpi. The *fgf* gene was highly expressed from 24 to 48 hpi, while *hoar* showed high expression from 0 to 12 hpi. During the transition from 12 to 24 hpi, *EONV_gp112* and *major early-transcribed protein 53* (*me53*) were highly expressed. *ChaB2* maintained high expression from 24 to 36 hpi.

Genes expressed highly at only a single time point often perform stage-specific functions. For example, at 12 hpi, *ubiquitin* and *ChaB2* were upregulated. By 24 hpi, genes such as *very late factor 1* (*vlf-1*), *late expression factor 2* (*lef2*), *glycoprotein 41* (*gp41*), and *24 kDa capsid protein* (*p24*) were induced. At 36 hpi, high expression was observed for *Vp39*, *lef5*, and *Bro-b*. At 48 hpi, a substantial number of very late genes were highly expressed, including auxiliary functional proteins (*Bro-a* and *P26b*), the viral structural protein *Odv-e25* (*occlusion-derived virus envelope protein 25*), regulators of DNA replication and gene expression (*alkaline exonuclease*, *nicotinamide riboside kinase 1* (*nrk-1*), and protein kinase 1 (*pk1*)), and the additional gene products *EONV_gp105* and *EONV_gp020*.

### 3.4. Transcriptional Dynamics of EcobNPV-QF4 in E. grisescens

**Figure 4:**
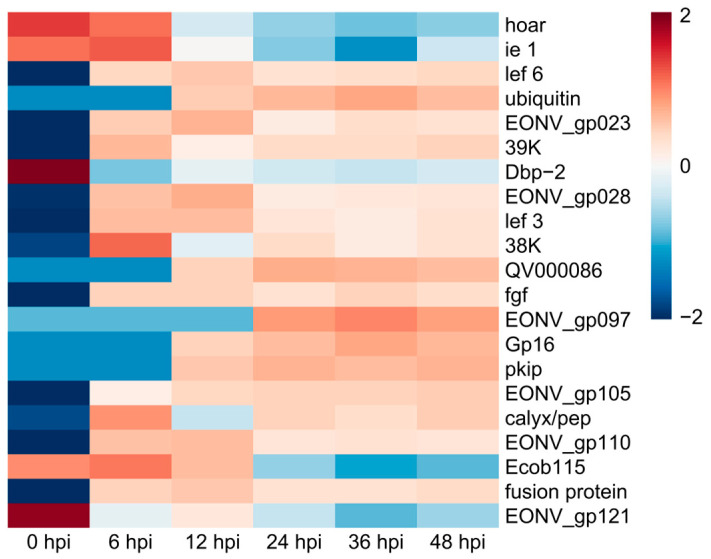
Heatmap of highly expressed genes (TPM > 20,000) across different time points in the EcobNPV-QF4. The heatmap depicts the expression dynamics (measured in TPM) of viral genes with peak expression exceeding 20,000 TPM at any sampled time point (0, 6, 12, 24, 36, 48 h post-infection, hpi) during infection of *E. grisescens* by the EcobNPV-QF4 strain. Expression values (log_2_(TPM + 1)) were Z-score normalized across time points for each gene.

We next examined the transcriptional dynamics of EcobNPV-QF4 under the same experimental conditions. Transcriptional analysis of EcobNPV-QF4 at 0, 6, 12, 24, 36, and 48 hpi revealed a consistent global expression trend across time points (Figure S5 and Table S4). Using a threshold of TPM > 20,000 to define high expression, we identified 48 viral genes that were highly expressed at one or more time points during infection (Table S5). The expression patterns fell into distinct temporal clusters (Figure 4). A subset of genes, including *fgf*, exhibited sustained high expression from 6 to 48 hpi. Others showed phase-specific expression: for instance, *ie1* and *Ecob115* were enriched early (0–12 hpi), while *gp16* and *pkip* were upregulated later (24–48 hpi). Notably, *lef6* displayed a biphasic expression pattern, with peaks at 6–12 hpi and again at 48 hpi.

Several genes were highly expressed only at discrete time points, indicating tight temporal regulation. These included *Dbp-2* (0 hpi), *calyx/pep*, *38 K*, and *39 K* (6 hpi), as well as *ubiquitin* and *Eonv_gp097* (36 hpi). Genes such as *hoar*, *lef3*, *fusion protein*, *EoNV_gp023*, *EoNV_gp028*, and *EoNV_gp110* were specifically upregulated during the early to mid-phase of infection (0–12 hpi). Additionally, *EoNV_gp105* and the functionally uncharacterized *QV000086*, which possesses an early promoter, were highly expressed from 12 hpi onward.

### 3.5. Distinct Expression Profiles of Functional Gene Categories in EcobNPV-QV and EcobNPV-QF4

**Figure 5:**
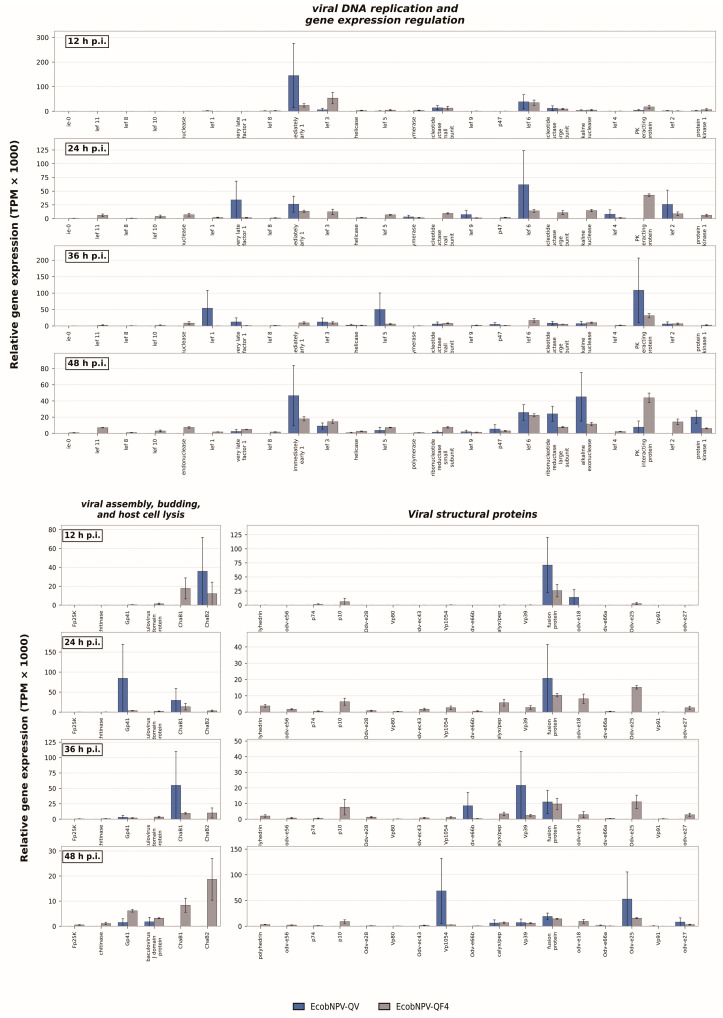

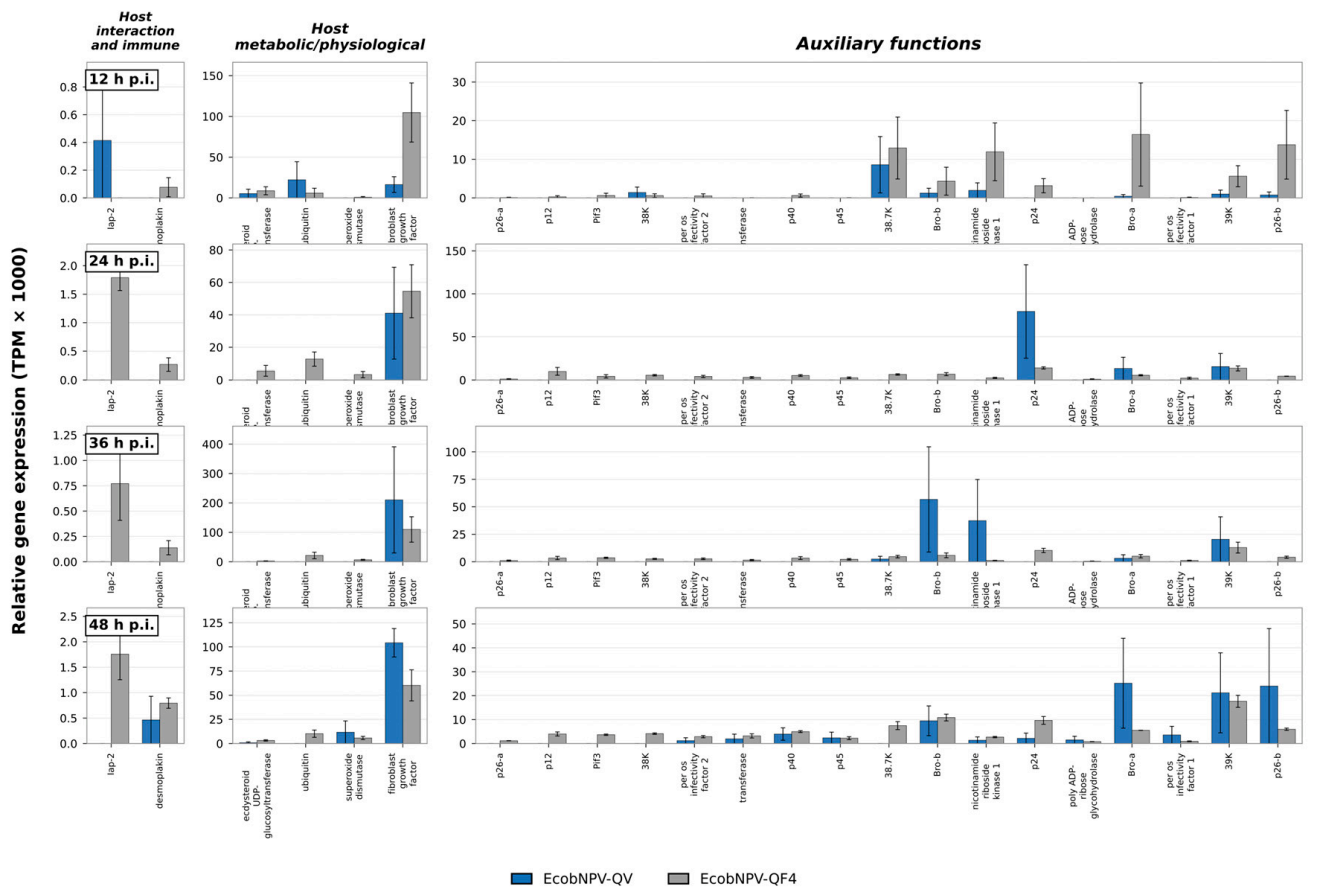
Functional classification and expression profiles of viral genes of EcobNPV-QV and EcobNPV-QF4. Each subplot is marked with gene functions. Blue bars stand for EcobNPV-QV, and gray bars for EcobNPV-QF4, showing mean TPM at 12, 24, 36, and 48 hpi. Time points (0, 2, 6 hpi) were excluded due to inadequate viral reads.

Next, we examined the relative expression patterns of several functionally grouped gene sets in the two EcobNPV strains. The expression profiles of each gene set in EcobNPV-QV were systematically compared to those of the same group in EcobNPV-QF4 (Figure 5). We restricted the analysis to 12–48 hpi, when viral replication is active because viral reads at earlier time points were insufficient for robust comparison (Tables S2 and S4). Viral genes were categorized into five functional groups based on their known roles in the baculovirus life cycle and genome annotation: structural proteins, DNA replication and transcription-related proteins, host interaction and immune modulation factors, proteins involved in virion assembly and budding, and auxiliary proteins. This classification follows commonly used schemes in baculovirus genomic studies [8,23]. Expression levels were quantified using TPM values. Figure 5 presents a series of horizontal bar charts depicting the expression patterns of genes from each functional category at 12, 24, 36, and 48 hpi for both strains. Comparative analysis revealed distinct expression patterns between the two strains. Overall, the number of functionally active genes (i.e., those with detectable expression, TPM > 1) in EcobNPV-QF4 exceeded that in EcobNPV-QV at each time point. However, for specific genes that were expressed in both strains, the highest TPM value was often observed in EcobNPV-QV.

Below, we highlight key differences observed in the viral structural protein category as a representative example, while patterns for the remaining categories are detailed in the Supplementary Materials. For EcobNPV-QV, only the *fusion protein* (*F*) and *odv-e18* were highly expressed at 12 hpi, and their expression levels surpassed those in EcobNPV-QF4. At 24 hpi, the *fusion protein* remained the sole highly expressed structural gene in EcobNPV-QV. In contrast, EcobNPV-QF4 displayed coordinated expression of multiple structural genes beginning at 24 hpi, a pattern that remained consistent thereafter. This included envelope proteins (*fusion protein*, *odv-e56*, *Odv-ec43*, *Odv-e28*, *Odv-e66a*, *odv-e18*, *odv-e27*, *Odv-e66b*, *Odv-e25*, *p74*), nucleocapsid proteins (*Vp1054*, *Vp39*), and occlusion-derived matrix proteins (*p10*, *calyx/pep*, *polh*). By 48 hpi, the structural gene repertoire of EcobNPV-QV expanded to include *Vp1054*, *caly/pep*, *Odv-e25*, and *odv-e27.* Similar patterns, where EcobNPV-QF4 activated a broader repertoire of functionally related genes earlier and more synchronously, while EcobNPV-QV showed delayed and more restricted expression of individual genes, were also observed in the other functional categories, including viral DNA replication and gene regulation, host interaction and immune modulation, viral assembly/budding/lysis, and auxiliary functions.

### 3.6. Rank-Based Identification of Differentially Regulated Genes

The functional category analysis revealed that EcobNPV-QF4 generally activates a broader repertoire of genes than EcobNPV-QV. However, because absolute expression levels differ substantially between strains (due in part to differences in infection progression rates), direct comparisons of TPM values can be confounded. To circumvent this limitation, we employed a rank-based comparison method [17], ranking each viral gene (1–131) by its TPM value within each sample. A rank difference of >30 positions between strains defined significant up- or down-regulation. The number of genes significantly upregulated in EcobNPV-QF4 relative to EcobNPV-QV increased during infection: 4 (0 hpi), 42 (6 hpi), 57 (12 hpi), 81 (24 hpi), 69 (36 hpi), and 49 (48 hpi) (Table S6). No genes were consistently downregulated across all time points.

To validate these findings, we performed complementary DESeq2 analysis on one-to-one orthologous ORFs at time points with sufficient viral read depth. Of the 345 rank-selected genes, 96 (27.8%) were statistically supported by DESeq2 (adjusted *p* < 0.05, |log_2_FoldChange| ≥ 1; Table S6). The remaining genes corresponded to early time points (0–6 hpi) where viral reads were negligible, or were excluded by DESeq2 independent filtering due to low counts or model instability. Thus, while DESeq2 provided robust parametric support for later infection stages, the rank-based approach remained essential for sparse asynchronous data where standard count-based methods were underpowered.

Table 2 lists genes that were consistently upregulated in EcobNPV-QF4 relative to EcobNPV-QV across multiple time points. These include genes associated with viral structure (*polh*, *p10*, *calyx/pep*); auxiliary functions (*p40*, *Pif3*, *p12*); viral assembly, budding, and host cell lysis (*bjdp*); and viral DNA replication and gene expression regulation (*lef8*), alongside others with unknown function. Notably, ten genes maintained a high rank from 12 hpi onward: *viral capsid associated protein*, *EONV_gp091*, *polh*, *p10*, *EONV_gp059*, *EONV_gp070*, *Pif3*, *EONV_gp035*, *EONV_gp107*, and *p12*.

**Figure 6:**
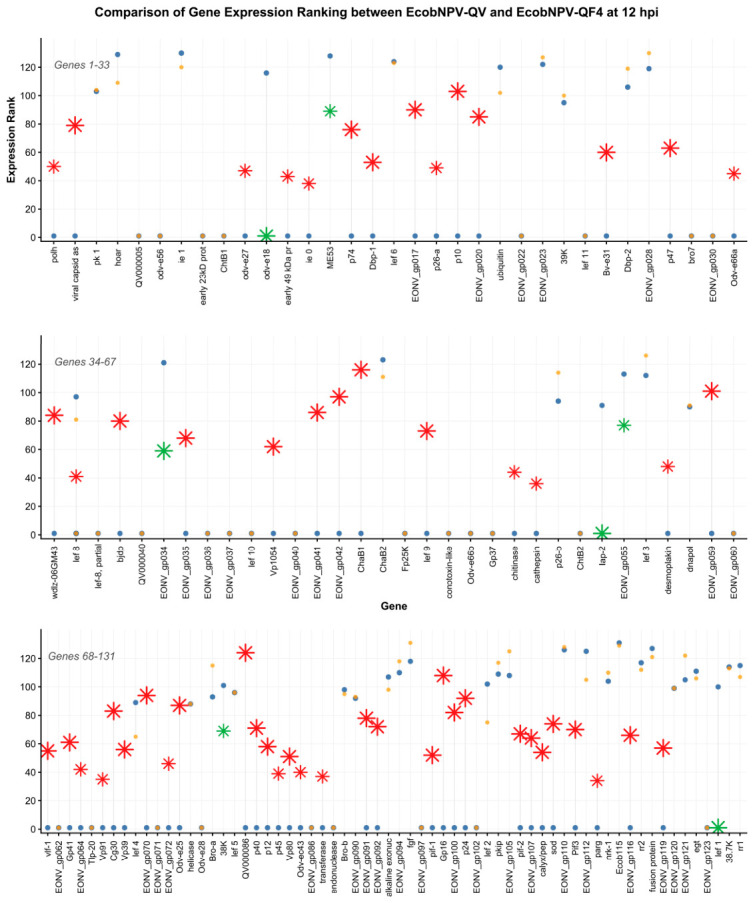
Comparison of gene expression ranking between EcobNPV-QV and EcobNPV-QF4 strains at 12 hpi. For each viral gene, its expression level (TPM) was compared against all other viral genes at the same time point to determine a relative expression rank within each strain. The scatter plot compares the rank of each gene in the EcobNPV-QV strain (blue data points) against its rank in the EcobNPV-QF4 strain (orange data points) at 12 hpi. Gene names are annotated on the x-axis. A red asterisk (*) indicates genes whose expression rank is significantly higher in QF4 than in QV (rank difference > 30), while a green asterisk (*) indicates genes with a significantly lower rank in QF4 (rank difference < −30).

At 12 hpi, EcobNPV-QF4 had already initiated robust late gene expression, as evidenced by high levels of *polh* and *p10*, whereas EcobNPV-QV appeared to be still undergoing the early-to-late transition with these genes remaining at low levels. This temporal divergence makes 12 hpi a critical time point for identifying genes whose accelerated expression in EcobNPV-QF4 may drive its faster infection cycle. To further explore strain-specific differences at this time point, we performed a comparative ranking of viral gene expression levels between the two strains. The analysis revealed that 57 genes were significantly upregulated in EcobNPV-QF4 (Figure 6). These genes belonged to multiple functional classes, including viral structural components, DNA replication and transcriptional regulators, and host interaction proteins, including 20 functionally uncharacterized genes. In contrast, only seven genes showed higher expression in EcobNPV-QV.

### 3.7. Time-Shift Correlation Analysis Between the Two Strains

**Figure 7:**
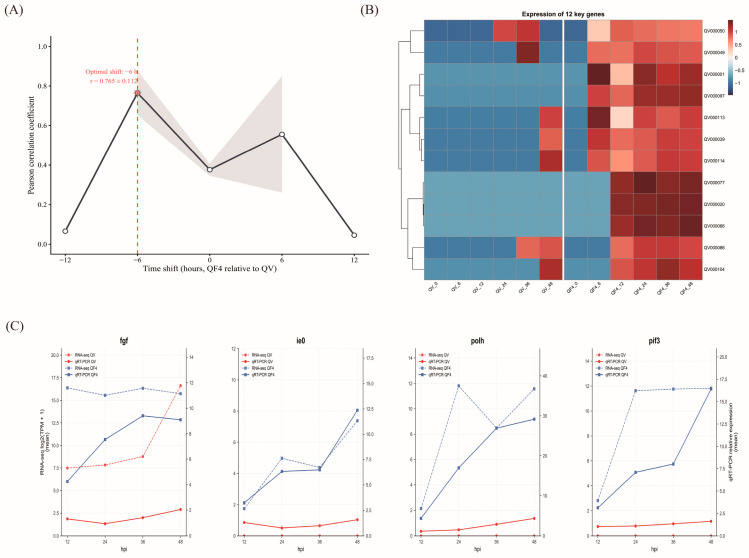
Temporal shift correlation and early highly expressed genes of EcobNPV-QF4 versus EcobNPV-QV. (**A**) Mean Pearson correlation between the transcriptomes of EcobNPV-QF4 and EcobNPV-QV across time offsets. The optimal shift of −6 h (dashed line) yielded the maximum correlation (*r* = 0.765). Shaded area, SE. (**B**) Expression heatmap of the top 12 genes significantly upregulated in EcobNPV-QF4 (fold change > 2) across all examined time points relative to EcobNPV-QV. Values are row-scaled log_2_(TPM + 1). Time points are grouped by strain (left: EcobNPV-QV; right: EcobNPV-QF4). (**C**) Validation of RNA-seq data by qRT-PCR. Comparison of expression patterns of four selected viral genes (*fgf*, *ie0*, *polh*, *pif3*) in EcobNPV-QV and EcobNPV-QF4 at 12, 24, 36, and 48 hpi, as measured by RNA-seq (dashed lines, left y-axis) and qRT-PCR (solid lines, right y-axis). Expression levels are presented as log_2_(TPM + 1) for RNA-seq data and relative expression values for qRT-PCR data.

To determine whether the global transcriptional program of EcobNPV-QF4 represents a temporally shifted pattern relative of EcobNPV-QV, we performed a time-shift correlation analysis across all shared viral genes. In this analysis, a negative shift indicates that the EcobNPV-QF4 expression profile was aligned with a later time point of EcobNPV-QV, suggesting an earlier transcriptional progression in EcobNPV-QF4. The correlation peaked at a shift of −6 h (mean *r* = 0.765, SE = 0.112), substantially exceeding the values observed under synchronous alignment (0 h: *r* = 0.377, SE = 0.030) and at positive shifts (+6 h: *r* = 0.556, SE = 0.294; ±12 h: *r* ≈ 0.05–0.07) (Figure 7A). Although the +6 h shift also yielded a moderate positive correlation, its relatively large associated standard error (SE = 0.294) indicated substantial variability among genes and therefore lower robustness than the −6 h peak. At the optimal −6 h shift, 88.2% of the analyzed genes exhibited positive correlations, demonstrating that the observed temporal advance was broadly distributed among viral genes rather than being driven by a small number of outliers. These observations indicate that the −6 h shift represents the optimal alignment.

To further assess the statistical robustness and gene-level consistency of the observed −6 h shift, we performed three complementary analyses. First, we determined the optimal shift for each individual gene (i.e., the shift at which its Pearson correlation reached the maximum). The distribution of gene-level optimal shifts showed that a combined 73.75% of genes exhibited their best alignment at negative shifts (−12 h: 37.5%; −6 h: 36.25%), whereas only 12.5% favored synchronous alignment (0 h) and 13.75% favored positive shifts (+6 h: 5.0%; +12 h: 8.75%). This indicates that the temporal advancement of EcobNPV-QF4 is broadly distributed across the viral genome rather than being driven by a small subset of genes (Figure S6A). Second, gene-level bootstrap resampling showed that −6 h was selected as the optimal candidate alignment in 71.8% of the 1000 iterations, whereas +6 h was selected in 28.2%. The bootstrap 95% confidence interval for −6 h was 0.543–0.946, which did not overlap with that for 0 h (0.319–0.436). In contrast, the confidence interval for +6 h was considerably wider (−0.091–1.000) and substantially overlapped with that for −6 h, indicating uncertainty in distinguishing the two candidate alignments (Figure S6B). Third, a paired *t*-test comparing the correlation coefficients of individual genes at −6 h versus 0 h revealed a significant difference (mean difference = 0.4295, 95% CI: 0.1694–0.6897, *t* = 3.359, df = 33, *p* = 0.002), confirming that the −6 h alignment yields statistically higher correlations than synchronous alignment at the individual gene level. Taken together, these analyses identify −6 h as the best-performing candidate alignment relative to synchronous alignment and support a tendency toward earlier transcriptional progression in EcobNPV-QF4.

To identify viral genes that may contribute to the enhanced virulence of EcobNPV-QF4, we compared gene expression levels between the two strains during the early stage of infection (0–12 hpi). Genes with a mean TPM > 1 in EcobNPV-QF4 and a fold change >2 relative to EcobNPV-QV were considered early-expressed genes. A total of 82 such genes were identified (Table S7), suggesting that their elevated expression in the early phase might facilitate viral replication and host manipulation, ultimately leading to higher mortality. A heatmap of the top 12 genes with the highest correlation under this optimal time shift is presented in Figure 7B, confirming their advanced expression in EcobNPV-QF4. Functional classification of these genes revealed five genes of unknown function (*QV000086*, *EONV_gp042*, *EONV_gp059*, *EONV_gp070*, *EONV_gp091*); three viral structural proteins (*calyx/pep*, *Gp16*, *polh*); two proteins involved in viral assembly, budding, and host cell lysis (*ChaB1*, *bjdp*); and one host immune modulation gene (superoxide dismutase). To validate the RNA-seq quantification, we examined the expression of four viral genes (*fgf*, *ie0*, *polh*, and *pif3*) by qRT-PCR at 12, 24, 36, and 48 hpi in EcobNPV-QV and EcobNPV-QF4 infected larvae and compared these results with RNA-seq data (Figure 7C). Importantly, the qRT-PCR results showed strong concordance with the RNA-seq rank-based expression patterns for all four tested genes, confirming that the observed differences are not artifacts of library preparation or sequencing and supporting the reliability of our transcriptomic analysis.

## 4. Discussion

*Ectropis grisescens* and *E*. *obliqua*, two morphologically similar geometrid moths, have long been misidentified as a single species in Chinese tea plantations. Following their taxonomic distinction through morphological and molecular characterization [24], subsequent research revealed that *E. grisescens* exhibits a larger body size, faster developmental rate, higher reproductive potential [25], and a broader geographical distribution [26] compared to *E. obliqua*. To enhance the biological control of the more widespread and damaging *E. grisescens*, a highly virulent strain, EcobNPV-QF4, was previously isolated. Comparative bioassays on third-instar larvae demonstrated that EcobNPV-QF4 achieved significantly higher genomic copy numbers and a faster replication rate in *E. grisescens* than the reference strain EcobNPV-QV [5]. Genomic analysis identified a large-scale inversion within the *homologous repeat regions* (*hrs*) between the two strains [5]. EcobNPV-QF4 exhibited significantly higher larval mortality than EcobNPV-QV at all evaluated time points, with the mortality gap emerging at 10 dpi and widening substantially by 16 dpi. Notably, all EcobNPV-QF4-infected pupae failed to emerge, whereas the majority of EcobNPV-QV-treated pupae survived, confirming that the virulence advantage of EcobNPV-QF4 extends to the pupal stage. To characterize the transcriptional dynamics driving the virulence disparity, we conducted a time-course transcriptomic profiling of viral gene expression in *E. grisescens* at 0, 2, 6, 12, 24, 36, and 48 hpi following oral infection with EcobNPV-QV and EcobNPV-QF4 and compared the expression programs between the two strains. EcobNPV-QF4 exhibits a globally accelerated transcriptional program, with the infection timeline compressed by approximately six hours relative to EcobNPV-QV. This acceleration is evident across multiple functional modules, consistent with the hypothesis that the enhanced virulence of EcobNPV-QF4 may involve coordinated reprogramming of viral gene expression.

Distinct gene expression profiles were observed between the strains (Figures S4 and S5). Analysis of highly expressed genes revealed that EcobNPV-QV exhibits a canonical, phased expression pattern, in which early genes (*ie1*, *me53*) [27,28] peaked prior to late genes (*lef6*, *fgf*, *fusion protein*) [8,29,30,31] (Figure 3). This phased cascade is consistent with the canonical baculovirus transcriptional program established across the family *Baculoviridae* [32]. In contrast, the EcobNPV-QF4 demonstrated a more synchronized and accelerated program (Figure 4). While strain-specific differences in replication kinetics have been reported in other baculoviruses, such as *egt* deletion-mediated accelerated killing in SfMNPV variants [33], the coordinated temporal compression of the entire gene expression cascade observed here has not, to our knowledge, been previously documented. This pattern suggests a more synchronized induction of a broader functional repertoire, which may affect multiple stages of infection concurrently.

Globally, the entire transcriptional program of EcobNPV-QF4 was advanced by approximately six hours relative to that of EcobNPV-QV (Figure 7). This temporal shift suggests an accelerated transcriptional timeline and may be linked to earlier downstream infection events, although midgut invasion, systemic dissemination, and virion assembly were not directly measured in this study. Notably, a comparable temporal offset has been reported in AcMNPV between midgut and cell lines, suggesting that modulation of transcriptional timing may be a general adaptive strategy among baculoviruses [17]. However, the 6 h resolution of our sampling interval precludes precise estimation of the true temporal offset and its confidence interval. Future studies with higher temporal resolution (e.g., 2 h intervals) would be needed to refine this estimate. This accelerated program involves the synchronized induction of a broader functional repertoire, optimizing multiple stages of infection concurrently; for example, genes involved in midgut escape (*fgf*), viral DNA replication (*lef3*, *pkip*), and occlusion body formation (*polh*, *p10*) are co-expressed earlier than in EcobNPV-QV. This coordinated strategy contrasts with the peak-focused, sequential expression pattern of EcobNPV-QV.

The enhanced virulence of EcobNPV-QF4 appears to be finely tuned to the biphasic replication cycle of baculoviruses [15]. Perhaps most strikingly, *fgf* was highly expressed in EcobNPV-QF4 throughout the infection cycle (6–48 hpi) (Figure 4), whereas its expression in EcobNPV-QV was restricted to 24–48 hpi (Figure 3). The earlier and sustained expression of *fgf* in EcobNPV-QF4 may contribute to this barrier-remodeling process and could potentially facilitate more rapid systemic dissemination [30]. An analogous function has been described for the enhanced midgut escape attributed to elevated *fgf* expression in AcMNPV-infected *T. ni* larvae [17]. In that system, *fgf* activates metalloproteases and caspases to degrade the basal lamina, a critical barrier for virus exit from the midgut [13,31,34]. Similarly, we propose that the early and persistent *fgf* expression in EcobNPV-QF4 may facilitate faster escape from the midgut epithelium [23,35], potentially allowing the virus to establish systemic infection at an earlier time point. However, this hypothesis is based solely on temporal correlations and requires direct functional validation.

EcobNPV-QF4 exhibits significantly accelerated genome replication compared with EcobNPV-QV, as evidenced by consistently elevated viral DNA copy numbers across all examined time points (0–48 hpi) [5]. This raises an important alternative interpretation: the apparently “earlier” or “stronger” transcriptional program in EcobNPV-QF4 may be partially driven by increased template availability, rather than solely reflecting intrinsic differences in transcriptional regulation. The concurrent early expression of *lef3* and *pkip* (Figure 4), both of which contribute to viral DNA replication and the production of BV following primary midgut infection [36,37], is consistent with an earlier mobilization of the replication machinery in EcobNPV-QF4 but does not directly demonstrate accelerated DNA replication kinetics. In baculovirus-infected cells, *lef3* is a single-stranded DNA-binding protein essential for origin-dependent DNA replication [36], and *pkip* interacts with *pk 1* to regulate late gene expression [37]. Their earlier co-expression in EcobNPV-QF4 may indicate earlier activation of a replication and BV-associated expression module, which could support earlier BV production. This pattern mirrors observations in AcMNPV-infected *T. ni* midgut, where genes associated with BV production (*gp64*, *p6.9*, *vp39*) are prioritized over occlusion body genes during the primary infection [17]. The acceleration extends to the very late phase: *polh* and *p10* were already highly expressed in EcobNPV-QF4 by 12 hpi, whereas they remained near baseline in EcobNPV-QV even at 24 hpi. This precocious activation of occlusion-related genes is compatible with the notion that EcobNPV-QF4 may compress the overall infection timeline.

Beyond these known regulatory and structural components, our comparative analysis also highlighted several uncharacterized genes that may constitute novel virulence determinants in this system. Among these, *EONV_gp091* and *QV000086* were consistently identified across multiple analyses (Figure 3 and Figure 4, Table 2), raising the possibility that they may represent novel virulence-associated factors specific to the EcobNPV lineage; however, functional characterization is required to establish their roles. *Hrs* function as both viral DNA replication origins and transcriptional enhancers [38]. The *ie1* serves as the master transcriptional regulator of baculoviruses and activates early gene transcription [39,40]. Inversion of the *hr* regions may alter the spatial relationship between *hr* elements and their target promoters, thereby modulating *ie1*-mediated transactivation of adjacent early genes. This rearrangement may also influence the efficiency of *hr*-dependent replication initiation. Collectively, we hypothesize that the *hr1*–*hr3* inversion in EcobNPV-QF4 may synergistically enhance both replication and transcriptional capacity relative to EcobNPV-QV. In addition to the *hr1*–*hr3* inversion, EcobNPV-QF4 harbors a duplication of the *rr2* [5], which may increase dNTP availability and support accelerated DNA replication during the late phase of infection [41]. However, *rr2* transcripts were only significantly elevated at 24 and 48 hpi, suggesting that the early transcriptional acceleration is primarily driven by the *hr* inversion rather than the *rr2* duplication. Nevertheless, this model is speculative and requires direct experimental testing, including assays for *ie1* binding affinity, targeted mutagenesis, and reciprocal reconstruction of the inverted region.

In addition to these viral-centric mechanisms, our previous study revealed that EcobNPV-QF4 infection induced more pronounced downregulation of host immune-related genes relative to EcobNPV-QV, including *caspase-8* and *PI3K* regulatory subunit alpha, and caused substantial alterations in amino acid metabolism (folate, fructose/mannose, and thiamine metabolism) and ribosome biogenesis pathways [42]. These host-centric changes suggest that QF4 more effectively suppresses host immune responses and reprograms host metabolism, thereby creating a more permissive environment for viral replication. Thus, the enhanced virulence of EcobNPV-QF4 likely reflects a dual strategy: an intrinsically accelerated viral transcriptional program (as demonstrated by our time-shift analysis) combined with more effective host immune evasion and metabolic reprogramming [42]. These mechanisms are not mutually exclusive but may act synergistically.

This study presents the first systematic analysis of viral gene expression profiles of two strains across the whole body of *E. grisescens*, comparing their expression patterns through multiple approaches. However, several limitations should be acknowledged. The use of whole larva samples may obscure fine-scale temporal dynamics and the distinction between primary infection and systemic spread, and the time-course analysis, based on seven sampling time points with intervals ranging from 2 to 12 h, although adequate for capturing global transcriptional trends, cannot rule out rapid, transient fluctuations between adjacent sampling points. Disentangling the relative contributions of accelerated DNA replication kinetics and intrinsic transcriptional regulation to the observed expression differences would require normalization of transcript levels to viral genome copy number at single-cell or single-genome resolution, a technically demanding undertaking currently precluded by the asynchronous nature of in vivo infection and the absence of a susceptible cell line for synchronous EcobNPV infection. Additionally, RNA sequencing data were collected only up to 48 hpi, whereas virulence assays were followed over a much longer duration, so this study is inherently limited to early infection events and cannot provide a comprehensive picture of the late-stage pathogenic cascades that ultimately determine host mortality. Developing a susceptible cell line for EcobNPV, combined with targeted functional genomics, is essential for testing causality between genomic variants, transcriptional reprogramming, and virulence enhancement and may ultimately guide the rational design of improved baculovirus insecticides for tea plantation pest management.

## 5. Conclusions

**Figure 8:**
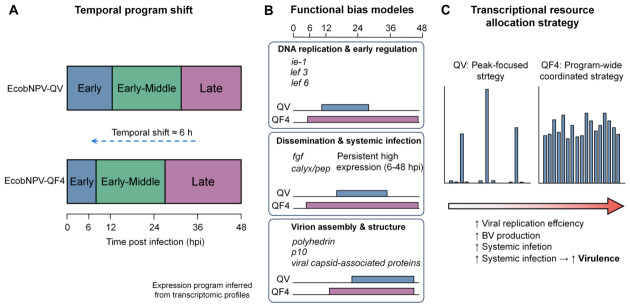
Proposed model of accelerated and broadened gene expression program underlying enhanced virulence of EcobNPV-QF4 in *E. grisescens*. (**A**) EcobNPV-QV shows sequential early /middle /late phases; EcobNPV-QF4 shows an accelerated early phase and earlier late-phase onset. The global transcriptional program of EcobNPV-QF4 is advanced by ~6 h relative to EcobNPV-QV. (**B**) EcobNPV-QF4 upregulates replication (*ie-1*, *lef-3*, *lef-6*), dissemination (*fgf*, *calyx*/PEP), and assembly genes (*polh*, *p10*, *viral capsid-associated protein*) earlier and for a longer duration. *fgf* is highly expressed at 6–48 hpi in EcobNPV-QF4 but delayed in EcobNPV-QV. (**C**) EcobNPV-QV shows a peak-focused pattern (few genes highly expressed at specific times); EcobNPV-QF4 shows a broad, coordinated pattern (many genes moderately but persistently expressed across stages).

This study delineates the first in vivo transcriptional landscape of EcobNPV in *E. grisescens*. Our results support a working model in which the enhanced virulence of EcobNPV-QF4 is associated with a temporally advanced gene expression program. It is proposed that this accelerated program may facilitate earlier midgut escape and more efficient systemic dissemination, potentially through the synchronized expression of genes involved in viral DNA replication, BV production, and virion assembly. However, this proposed accelerated transcriptional program is a working model that requires rigorous experimental testing to establish causality. We further identified key viral genes whose differential expression profiles show correlations with strain-specific pathogenicity, including *fgf*, *lef3*, *polh*, *p10*, and the previously uncharacterized ORFs *EONV_gp091* and *QV000086*. These findings provide an initial molecular framework for further investigation of baculovirus–host interactions in this agriculturally important pest system (Figure 8). Moreover, they point to specific viral genes and regulatory modules as potential targets for the rational design and optimization of recombinant baculovirus-based biopesticides with improved speed of kill and overall efficacy against *E. grisescens*.

## Figures and Tables

**Table 1:** Primer sequences for quantitative qRT-PCR.

Target Gene	Type	Primers (5′–3′)
*fgf*	Host metabolic/physiological	F: AAGTGGCGGAGACACAATGG R: ATGATGAGGAGGAGGAGTAGGG
*ie0*	Viral DNA replication and gene expression regulation	F: ACTCTCATCCACCACCAATC R: AGCAATAACACCGCCCAAC
*polh*	Viral structural proteins	F: AAAAACGCCAAACGCAAG R: GTTAATCACCAAAAACACGTCC
*Pif3*	Auxiliary functions	F: GTATATTTTGTTGCTGTGCCTC R: CATTTAGTTTCGCCATCGTTC
*GAPDH*	Housekeeping	F: TCCCTCAGCGGCTTCCTT R: AACATCATTCCAGCGTCCACT

**Table 2:** Detailed characteristWics of continuously expressed genes (high-expression frequency ≥ 4) with alignment differences between the two virus strains.

Gene_id	Annotation	6	12	24	36	48	Promoter	Type
QV000002	*viral capsid associated protein*	+	+	+	+	+	None	Viral structural proteins
QV000097	*EONV_gp091*	+	+	+	+	+	E, L	Unknown
QV000001	*polh*	+	+	+	+	+	L	Viral structural proteins
QV000020	*p10*		+	+	+	+	L	Viral structural proteins
QV000066	*EONV_gp059*		+	+	+	+	E, L	Unknown
QV000049	*EONV_gp042*	+	+	+		+	E	Unknown
QV000077	*EONV_gp070*		+	+	+	+	L	Unknown
QV000039	*bjdp*	+	+	+	+		E, L	Viral assembly, budding, and host cell lysis
QV000087	*p40*	+	+	+	+		L	Auxiliary functions
QV000116	*Pif3*		+	+	+	+	E, L	Auxiliary functions
QV000042	*EONV_gp035*		+	+	+	+	E, L	Unknown
QV000112	*EONV_gp107*		+	+	+	+	None	Unknown
QV000088	*p12*		+	+	+	+	L	Auxiliary functions
QV000113	*calyx/pep*	+	+	+	+		L	Viral structural proteins
QV000016	*Dbp-1*	+	+		+	+	E	Viral DNA replication and gene expression regulation
QV000024	*EONV_gp023*	+		+	+	+	None	Unknown
QV000100	*EONV_gp094*	+		+	+	+	None	Unknown
QV000008	*early 23kD protein*	+		+	+	+	None	Viral DNA replication and gene expression regulation
QV000035	*lef 8*	+		+	+	+	None	Viral DNA replication and gene expression regulation

Notes: The symbol ‘+’ denotes genes with an inter-strain ranking difference > 30. Putative promoters and functional categorization into five classes were based on homology predictions from the literature. E, early promoter; L, late promoter.

## Data Availability

The data presented in this study are available in this article.

## Supplementary Materials

The following supporting information can be downloaded at: https://www.mdpi.com/article/10.3390/microorganisms14071599/s1, Figure S1. Genome-wide read coverage distribution of EcobNPV-QV and EcobNPV-QF4 at different post-infection time points.; Figure S2. Principal component analysis (PCA) of viral gene expression using all individual biological replicates; Figure S3. Sample-to-sample Pearson correlation heatmap of viral gene expression across EcobNPV-QV and EcobNPV-QF4; Figure S4: Expression levels of EcobNPV-QV genes in *Ectropis grisescens*; Figure S5: Expression levels of EcobNPV-QF4 genes in *E*. *grisescens*; Figure S6: Robustness validation of the 6 h temporal shift in the time-shift correlation analysis; Table S1: RNA-seq quality and read mapping statistics of transcriptome samples; Table S2: EcobNPV-QV genes in the order of their gene id in *E*. *grisescens* at selected times post infection; Table S3: Summary of Highly Expressed Genes (TPM > 20,000) in EcobNPV-QV; Table S4: EcobNPV-QF4 genes in the order of their gene id in *E*. *grisescens* at selected times post infection; Table S5: Summary of Highly Expressed Genes (TPM > 20,000) in EcobNPV-QF4; Table S6: Gene ranking positions in EcobNPV-QF4 and EcobNPV-QV at different hours post infection (hpi); Table S7: Early-expressed viral genes in EcobNPV-QF4 compared to EcobNPV-QV.

## Abbreviations

The following abbreviations are used in this manuscript:

AcMNPV	Autographa californica multiple nucleopolyhedrovirus
BV	Budded virus
Ct	Cycle Threshold
dpi	days post-infection
EcobNPV	Ectropis obliqua nucleopolyhedrovirus
fgf	fibroblast growth factor
Gp41	Glycoprotein 41
Hpi	Hours post infection
Hrs	Homologous repeat regions
Ie0	Immediate early gene 0
lef2	late expression factor 2
lef3	late expression factor 3
lef6	late expression factor 6
LT50	median lethal time
me53	major early-transcribed protein 53
nrk-1	nicotinamide riboside kinase 1
OB	Occlusion body
OBs	Occlusion bodies
ODV	Occlusion-derived virion
OGS	Official gene set
ORFs	Open reading frames
p24	24 kDa capsid protein
PCA	Principal component analysis
pif3	per os infectivity factor 3
pk1	protein kinase 1
polh	polyhedrin
RIN	RNA integrity number
rr2	small subunit of ribonucleotide reductase
TPM	Transcripts Per Million
vlf-1	very late factor 1
