# Supplementary material for: Comparative Gene Expression Patterns of Two EcobNPV Strains in Ectropis grisescens Revealed by Transcriptome Analysis
Source: Microorganisms. 2026 Jul 22;14(7):1599. doi: 10.3390/microorganisms14071599 (PMC13414177; doi:10.3390/microorganisms14071599)
Supplement: Supplementary file 1 [file microorganisms-14-01599-s001.zip › microorganisms-4383750-supplementary figure.pdf]

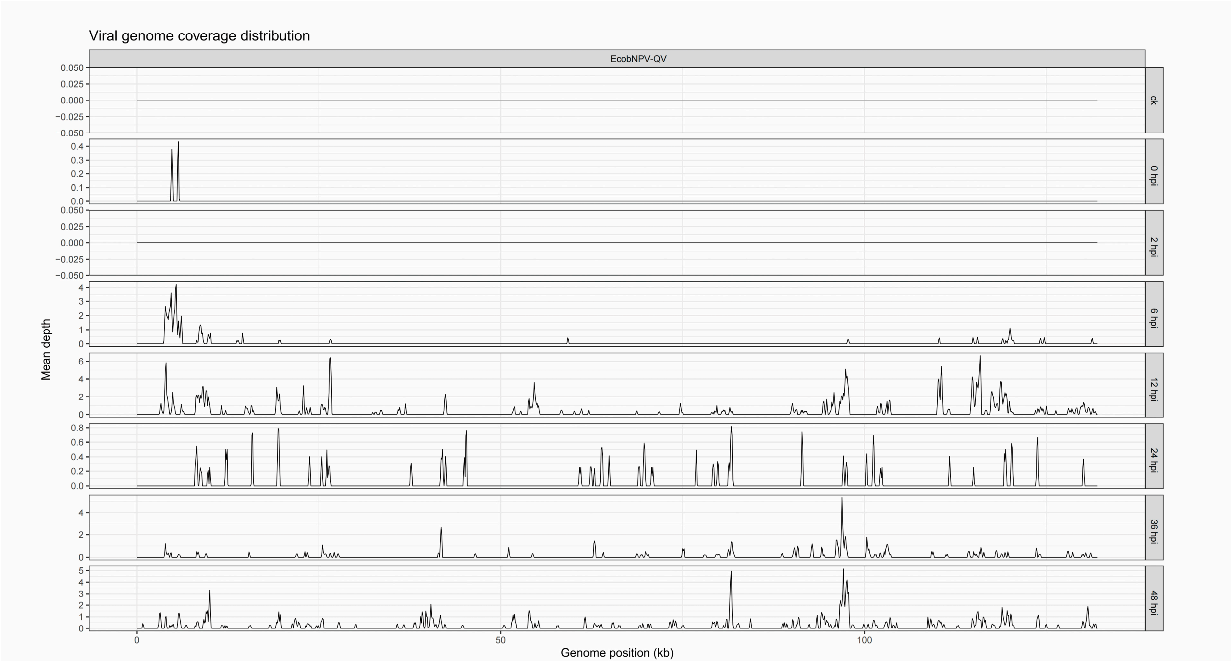

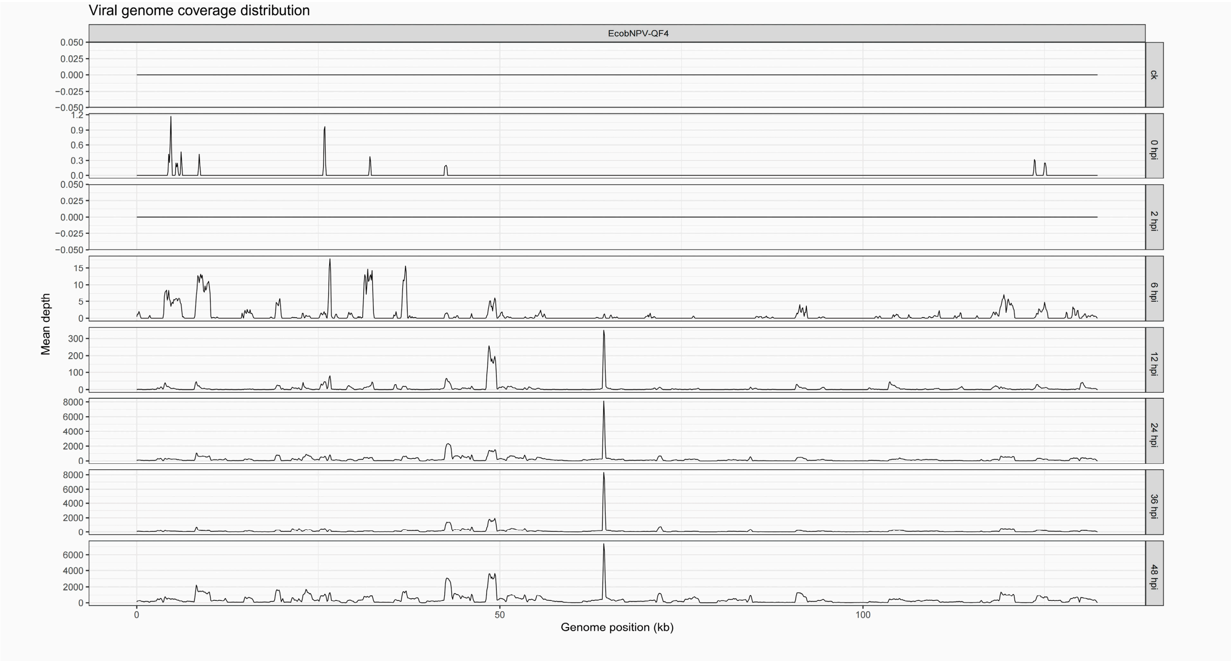

Figure S1. Genome-wide read coverage distribution of EcobNPV-QV and EcobNPV-QF4 at different post-infection time points.

The horizontal axis represents the physical position along the EcobNPV genome (unit: kilobase, kb), and the vertical axis denotes the mean sequencing depth of mapped RNA-seq reads. Each horizontal panel corresponds to an individual sampling time point (ck, 0, 2, 6, 12, 24, 36, 48 hpi) post oral infection of *Ectropis grisescens*. The peak signals indicate genomic regions with abundant transcriptional read accumulation, reflecting the dynamic transcriptional activation of viral genes over the infection cycle.

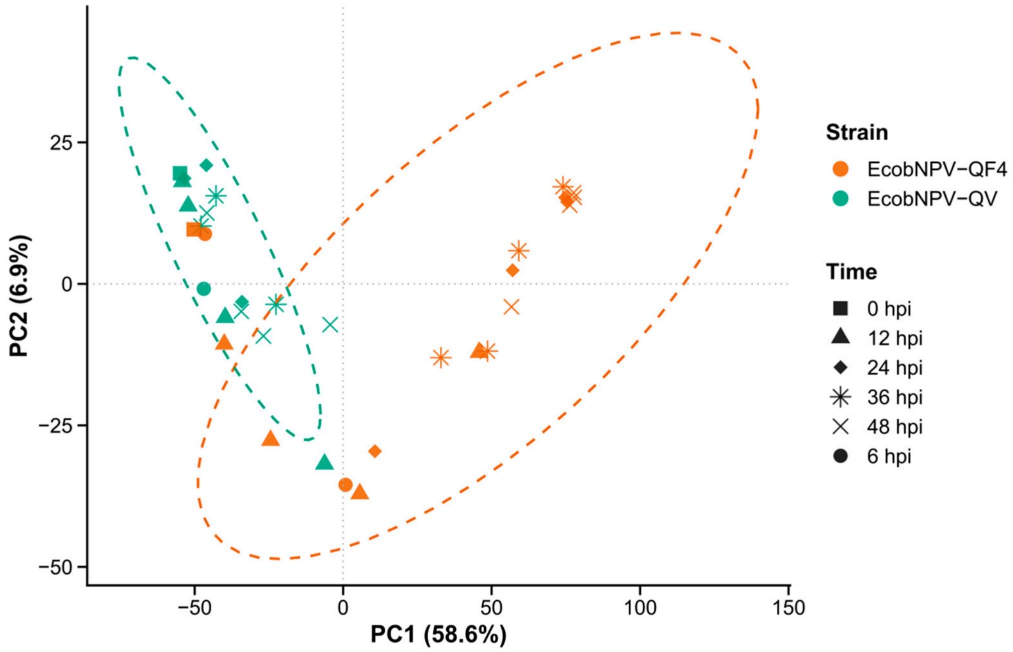

Figure S2. Principal component analysis (PCA) of viral gene expression using all individual biological replicates.

To evaluate the variability among replicates, PCA was conducted on the $\log_2(TPM+1)$ expression values of viral genes from all unmerged individual samples. The four replicate samples within each strain–time group cluster tightly, and the two strains are clearly separated along the PC1 axis, confirming high reproducibility across replicates and supporting the reliability of the mean-based analysis presented in the main text. Ellipses denote 95% confidence intervals for each strain.

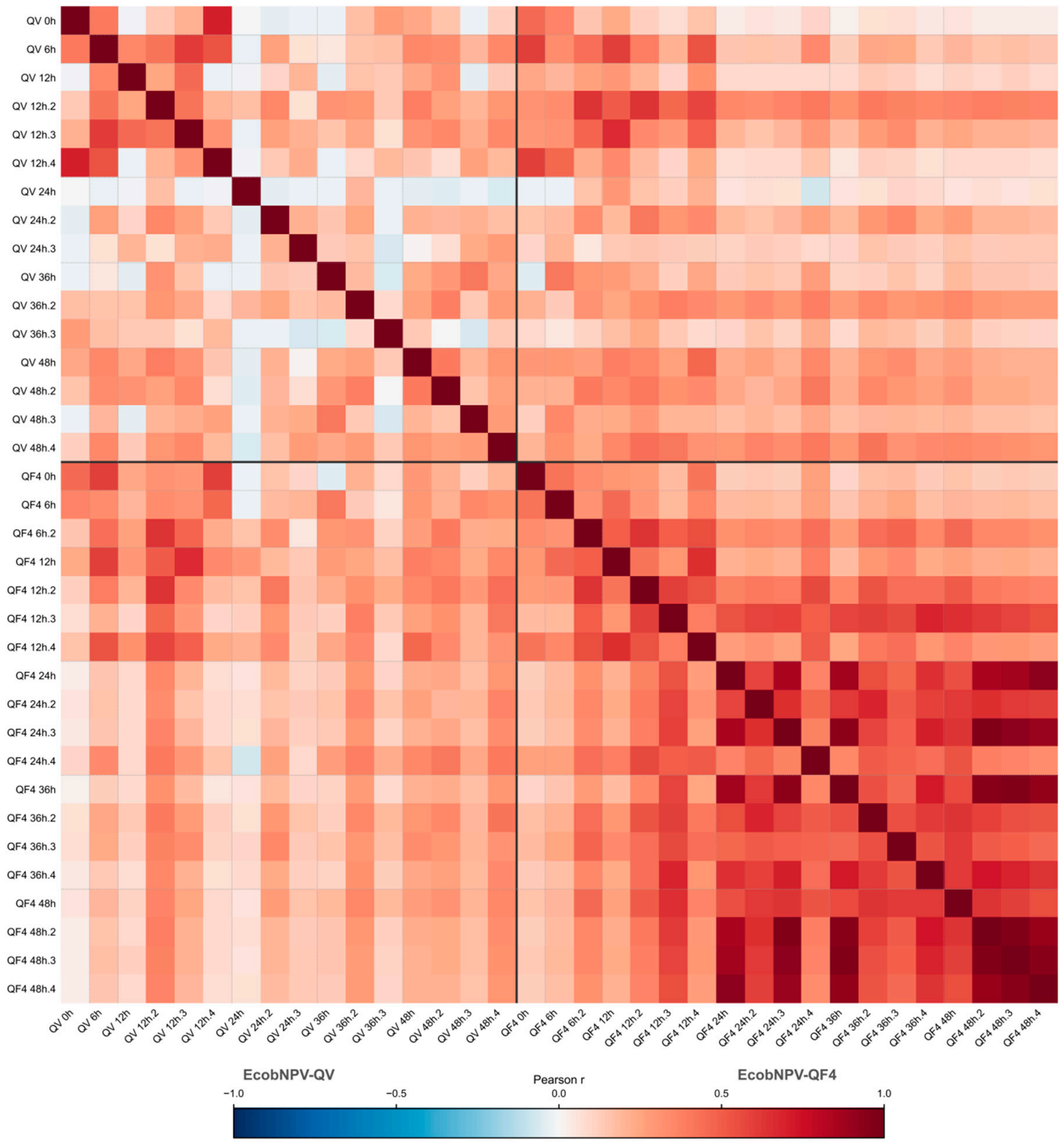

Figure S3. Sample-to-sample Pearson correlation heatmap of viral gene expression across EcobNPV-QV and EcobNPV-QF4.

The heatmap displays pairwise Pearson correlation coefficients of viral gene expression profiles ($\log_2$(TPM+1)) between RNA-seq samples collected at 0 ‒ 48 hpi. 21 samples had negligible viral RNA and were excluded from analysis. Correlation was calculated using all 131 viral genes. Samples are arranged sequentially by strain, time point, and biological replicate. The color scale from blue ($r = -1$) through white ($r = 0$) to red ($r = +1$) indicates the strength and direction of the correlation. Solid black lines separate the two viral strains; dotted gray lines separate different time points within each strain.

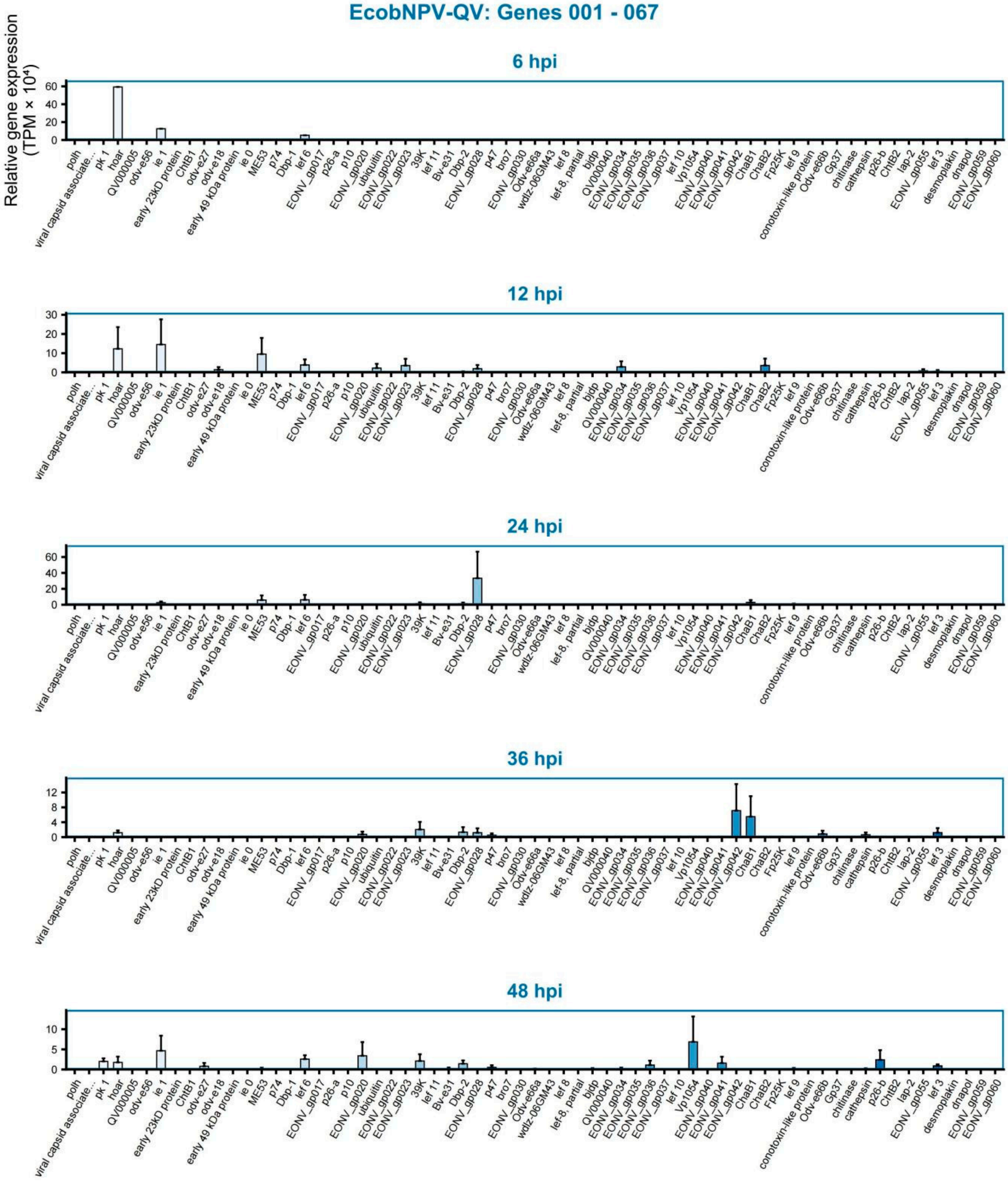
EcobNPV-QV: Genes 001 - 067
Relative gene expression (TPM × 10⁴)
6 hpi
12 hpi
24 hpi
36 hpi
48 hpi
polh
viral capsid associate...
pk 1
hoar
QV000005
odv-e56
ie 1
early 23KD protein
ChtB1
odv-e27
odv-e18
early 49 kDa protein
ie 0
ME53
p74
Dbp-1
lef 6
EONV_gp017
p26-a
p10
EONV_gp020
ubiquitin
EONV_gp022
EONV_gp023
39K
lef 11
Bv-e31
Dbp-2
EONV_gp028
p47
bro7
EONV_gp030
Odv-e66a
wdlz-06GM43
lef 8
lef-8, partial
bjdp
QV000040
EONV_gp034
EONV_gp035
EONV_gp036
EONV_gp037
lef 10
Vp1054
EONV_gp040
EONV_gp041
EONV_gp042
ChaB1
ChaB2
Fp25K
lef 9
conotoxin-like protein
Odv-e66b
Gp37
chitinase
cathepsin
p26-b
ChtB2
lap-2
EONV_gp055
lef 3
desmoplakin
dnapol
EONV_gp059
EONV_gp060

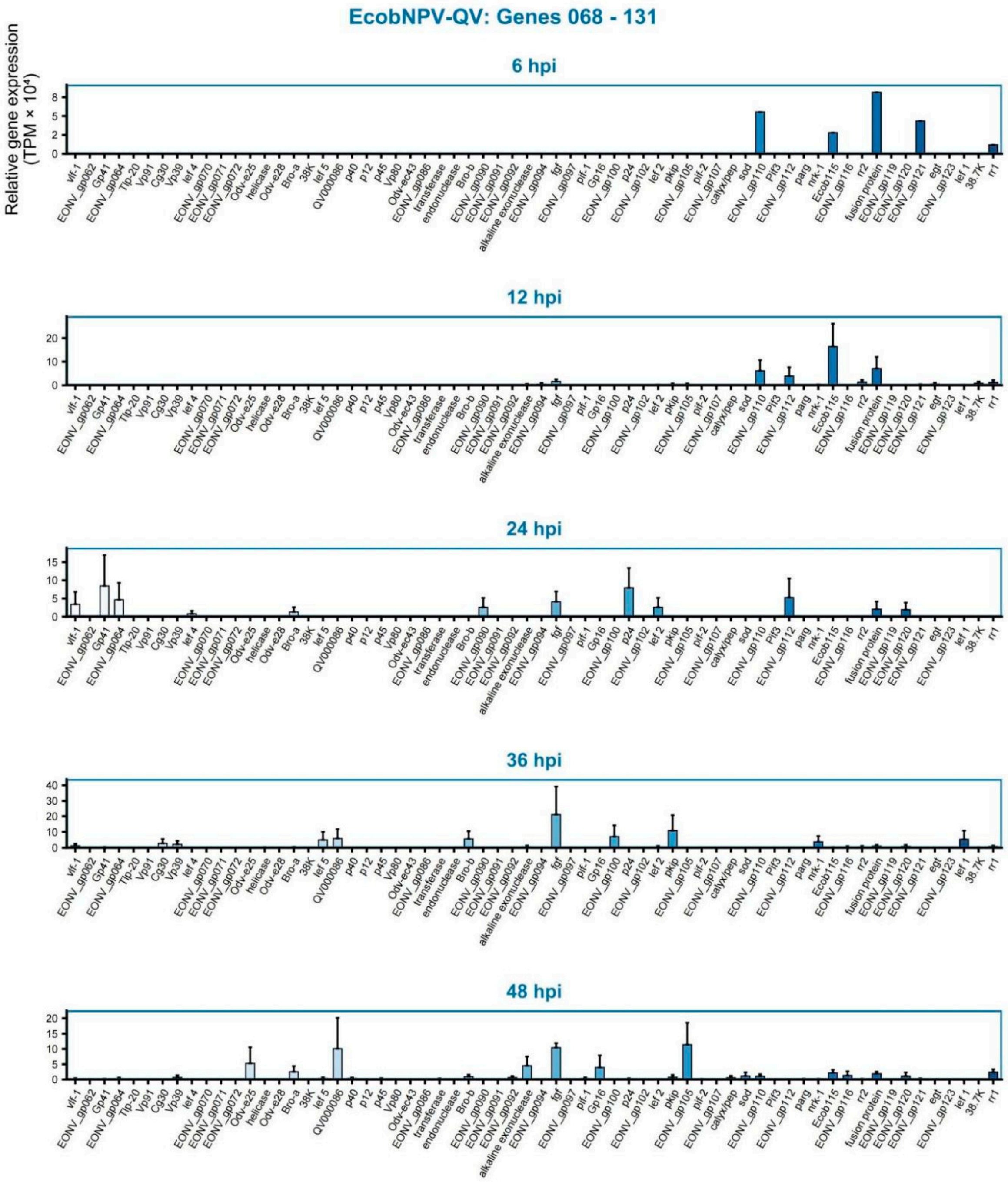

Figure S4. Expression levels of EcobNPV-QV genes in *E. grisescens*.

Gene expression is represented by the mean transcripts per million (TPM) values at each time point (error bars = SD; n = 4 biological replicates). TPM values were calculated by StringTie based on aligned viral reads for each gene.

EcobNPV-QF4: Genes 001 - 067

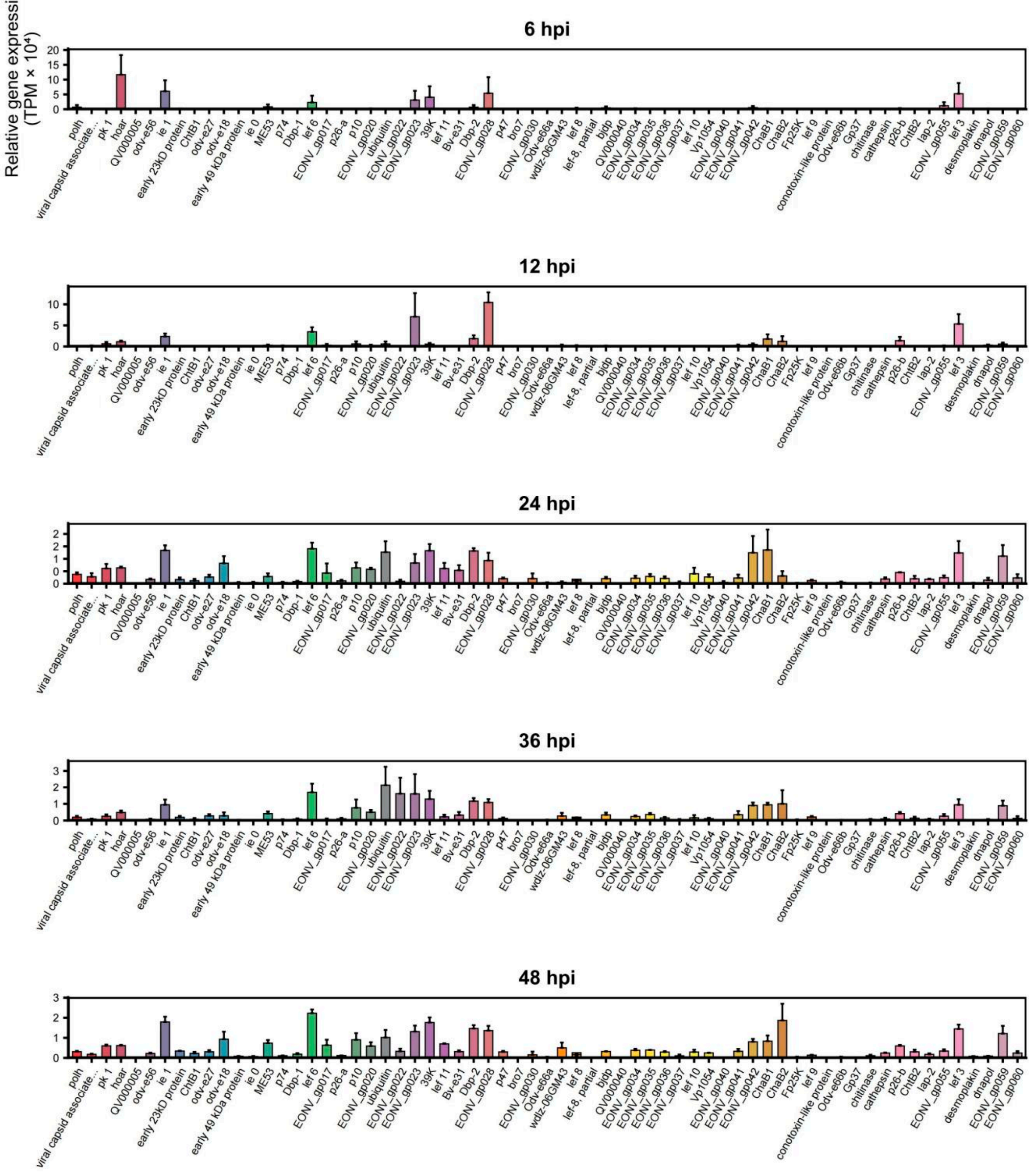

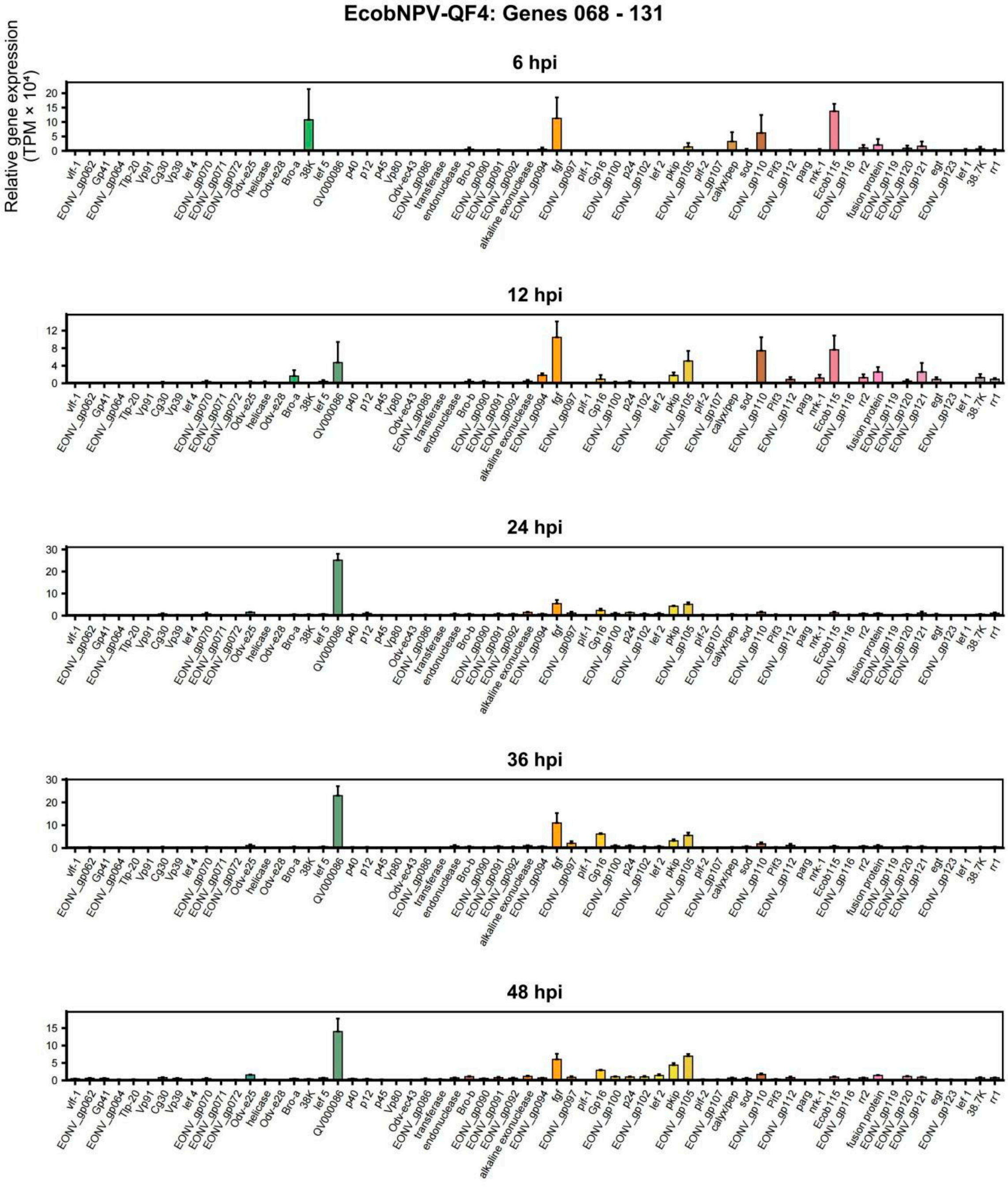

Figure S5. Expression levels of EcobNPV-QF4 genes in *E. grisescens*.

Gene expression is represented by the mean transcripts per million (TPM) values at each time point (error bars = SD; n = 4 biological replicates). TPM values were calculated by StringTie based on aligned viral reads for each gene.

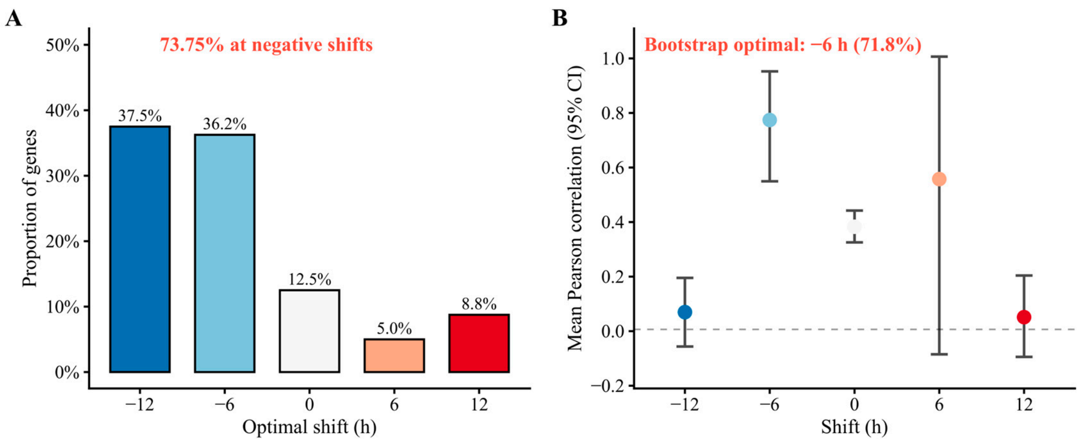

Figure S6. Robustness validation of the 6-hour temporal shift in the time-shift correlation analysis. (A) Distribution of gene-level optimal shifts (i.e., the shift at which the Pearson correlation coefficient between EcobNPV-QF4 and EcobNPV-QV expression profiles reached its maximum for each individual viral gene). Negative values indicate that the EcobNPV-QF4 expression profile aligns with a later time point of EcobNPV-QV, reflecting an earlier transcriptional progression in EcobNPV-QF4. (B) Mean Pearson correlation coefficients and their 95% confidence intervals for each shift estimated by bootstrap resampling (1,000 iterations with gene-level replacement).
